# Social inequalities and trends in pre-pregnancy body mass index in Swedish women

**DOI:** 10.1038/s41598-021-91441-7

**Published:** 2021-06-08

**Authors:** Christina E. Lundberg, Maria Ryd, Martin Adiels, Annika Rosengren, Lena Björck

**Affiliations:** 1 Department of Molecular and Clinical Medicine, Sahlgrenska Academy, University of Gothenburg, Gothenburg, Sweden; 2 Department of Medicine Geriatrics and Emergency Medicine/Östra, Region Västra Götaland, Sahlgrenska University Hospital, Gothenburg, Sweden; 3 School of Public Health and Community Medicine, Institute of Medicine, University of Gothenburg, Gothenburg, Sweden

**Keywords:** Medical research, Risk factors

## Abstract

Obesity rates in adolescence and young adulthood have increased in Sweden, reflecting global trends. To which extent this occurs across different socioeconomic strata has not been clarified. The aim of the present study was to investigate trends in social inequalities in body mass index (BMI) in young/mid-adulthood Swedish women. We obtained weight and height for all women aged 20–45 years, at their first registered pregnancy (< 12 weeks of gestation) in the Swedish Medical Birth Register 1982–2013 (1,022,330, mean age = 28.8 years), documenting education and county of residence. Trends in mean BMI and in the prevalence of BMI categories between 1982 and 2013 were estimated across education levels and geographical location. Overall, mean BMI increased from 22.7 kg/m^2^ (SD 3.2) to 24.3 kg/m^2^ (SD 4.4) between 1982 and 2013. Simultaneously, the prevalence of overweight and obesity (BMI ≥ 25 kg/m^2^) increased from 18.1 to 33.4% while that of moderate obesity (BMI ≥ 30 to < 35 kg/m^2^) and severe obesity (BMI ≥ 35 kg/m^2^) increased markedly from 3.4 and 0.4% to 7.4 and 3.1%, respectively. The prevalence of moderate and severe obesity more than doubled during the study period across all educational levels. In conclusion, BMI and moderate and severe obesity increased markedly among young/mid-adulthood Swedish women regardless of education with a widening gap between those with lower and higher education. These growing social inequalities in BMI are likely to cause a rising divide in serious health problems following early and long-lasting obesity.

## Introduction

The prevalence of obesity (BMI ≥ 30 kg/m^2^) overall, and particularly in children, adolescents and young adults, has risen dramatically worldwide in recent decades^1,2^, leading to multiple adverse health effects and increased financial costs for society^3–6^. Individuals who are obese during their childhood often remain obese as adults^7^, leading to more severe medical complications than among those who develop obesity during adulthood^8^. Compared to lean young men (18–25 years of age) and young/mid-adulthood (20–45 years of age) men and women, those obese already when young, face markedly elevated risks of developing early diabetes, stroke, venous thromboembolism and heart disease already at high normal BMI^9–14^.

There is a strong association between socioeconomic status and body weight, with an increasing risk of becoming overweight or obese in people with lower socioeconomic status^15–18^. In addition, individuals living in rural areas are more likely to be overweight or obese, even though this risk is attenuated when accounting for educational level^19^. Among adolescent Swedish men, social gradients in BMI and obesity widened between 1968 and 2005 with a more than threefold difference in severe obesity between men with the highest and the lowest parental education^16^.

However, there are limited similar data on temporal trends in the prevalence of moderate and severe obesity in young/mid-adulthood women. This pertains in particular as to whether socioeconomic differences in BMI have widened to the same extent as in young men aged 18–25 years, or whether there are differences between urban and rural areas, which has been described in middle-aged employed adults^15^. The aim of this study was therefore to investigate trends in social inequalities in mean BMI and in the prevalence of clinically relevant BMI categories ranging from underweight to severely obese in young/mid-adulthood women aged 20–45 years in Sweden.

## Methods

### Study design and population

The study population included all women aged 20–45 years registered at their first pregnancy in the Swedish Medical Birth Register (Birth Register) between 1 January 1982 and 31 December 2013 (n = 1,022,540). After exclusion of women with improbable reported height (< 140 cm or > 200 cm) or missing BMI (n = 210), the final study population comprised 1,022,330 women.

The Birth Register includes all births (99% coverage) in Sweden from 1973 and onwards, with height and weight registered since 1983, with the exception of 1990 and 1991 when no data on weight were collected. Until 1990, early pregnancy weight was calculated by subtracting gestational weight gain from the weight at delivery. Delivery weight was only recorded with two digits (e.g., a weight of ≥ 100 kg was registered as 99 kg). From 1992 and onwards, weight was measured and height was self-reported during the first antenatal visit (generally early in the first trimester in 90% of women), before any appreciable pregnancy-related weight gain. With previous studies describing weight gain during the first trimester as negligible, we used this first registered weight as a proxy for pre-pregnancy weight^20,21^. Visual inspection of annual body weight deciles showed a larger than expected increase in body weight between 1989 and 1992, coinciding with change in reporting. Therefore, the weight variable for 1982–1989 was adjusted by estimating the annual weight increase within deciles from 1992 to 2003, generating a practically linear result^13^.

The study procedure was approved by the Regional Ethical Review Board in Gothenburg, Sweden (Dnr:103-15). Because the data is coded (anonymous) inform consent was waived by Ethical Review Board for this study. The investigation conforms with the principles outlined in the Declaration of Helsinki.

### Social factors

Information on county of residence, educational level and county of birth was obtained by linking the Birth Register to the Longitudinal Integration Database for Health Insurance and Labour Market Studies (LISA) using the Personal Identification Number (PIN) assigned to all Swedish residents. Education (< 1% missing data) was categorized into three groups: lower (≤ 9 years), intermediate (10–12 years), and higher (> 12 years) level of education. Country of birth was categorized as born in Nordic countries, yes/no.

### Statistical analysis

The study period was dived into six periods: 1982–1988, 1989–1993, 1994–1998, 1999–2003, 2004–2008, and 2009–2013. Descriptive statistics including age-adjusted mean, standard deviation (SD), and percentage of weight, height and BMI were used to describe characteristics across the six periods, and stratified by education.

As the mean maternal age gradually increased over the study period (see Supplementary Fig. S1 online), age was adjusted to the age distribution in the period 2009–2013. Similarly, as the educational level gradually increased over the study period, the data were age-adjusted within each educational level. The age-adjusted mean BMI (weight (kg)/ height (m^2^)) with 95% confidence interval (CI) and prevalence of BMI (%) was calculated yearly for all women from 1982 to 2013, and stratified by educational level and county for the six periods. Prevalence of BMI was divided into eight clinically relevant categories, according to previous research from our group, demonstrating increased risk for several outcomes starting from lower than conventionally defined normal weight^10,11,13^: 15 to < 18.5 kg/m^2^, 18.5 to < 20 kg/m^2^, 20 to < 22.5 kg/m^2^, 22.5 to < 25 kg/m^2^, 25 to < 27.5 kg/m^2^, 27.5 to < 30 kg/m^2^, 30 to < 35 kg/m^2^, and 35 to < 60 kg/m^2^. All county data were adjusted within each county to the age distribution in the period 2009–2013, and adjusted for education.

Logistic regression was used to calculate age-adjusted prevalence ratios (PR) with 95% CI^22^ of overweight, moderate and severe obesity comparing lower versus higher educational level during each period. Corresponding PR was calculated for the six periods with the first period (1982–1988) as a reference, in total and stratified by educational level. Non overlapping CIs indicate whether the differences are significantly increasing or decreasing. Data management was performed using SAS version 9.4 (SAS Institute, Cary, NC, USA) and statistical analyses was performed in R version 4.0.2^23^. R was also used as image processing software, and the text in the figures was edited using Affinity Designer.

## Results

### Trends in BMI, overweight and moderate and severe obesity

**Figure 1:**
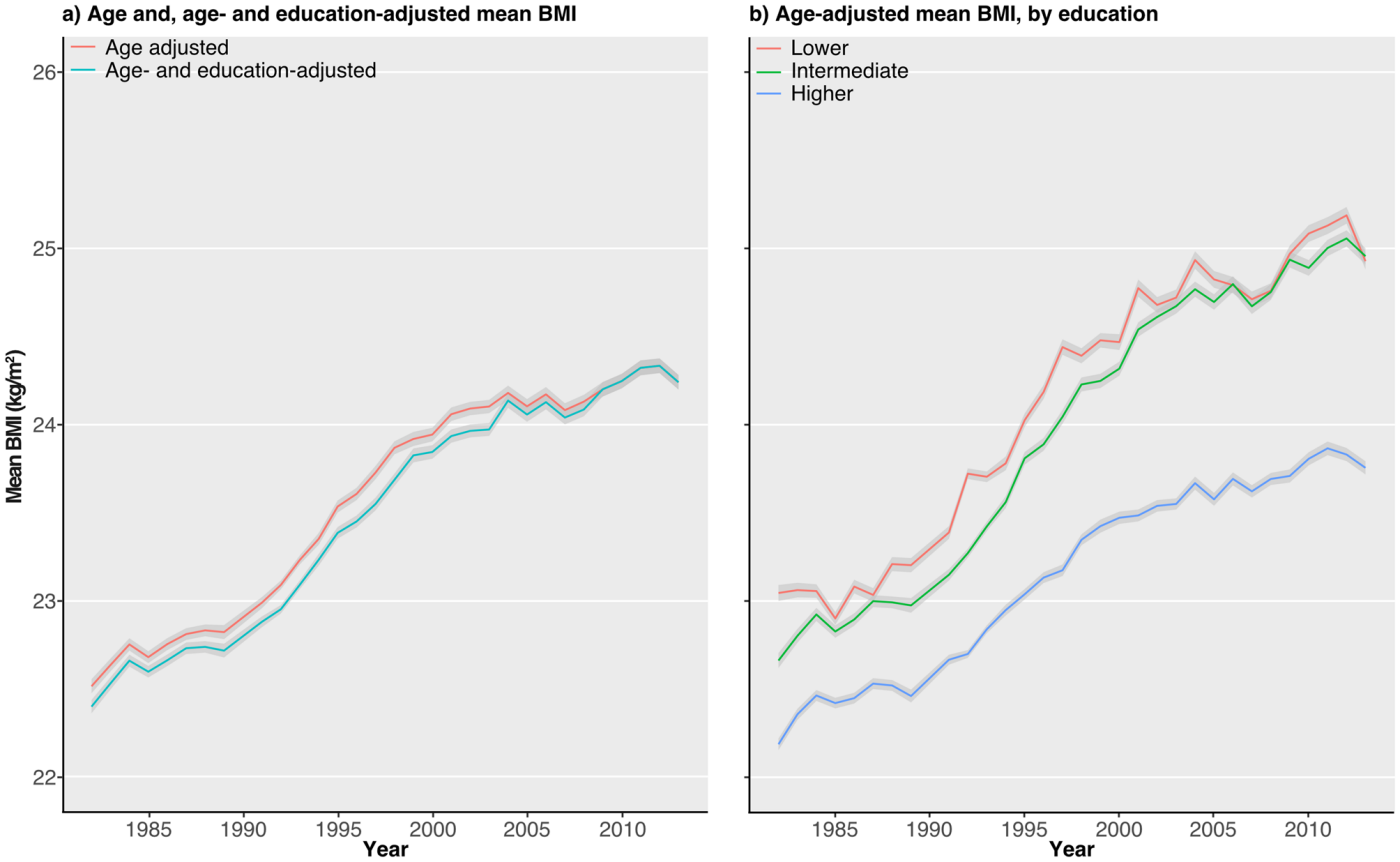
Trends in age-adjusted mean BMI. The lines show the yearly mean estimates and the shades show the 95% confidence intervals from 1982 to 2013. (**a**) Total cohort, age adjusted and age- and education adjusted, (**b**) by educational level adjusted for age. *BMI* body mass index.

The study cohort comprised 1,022,330 women aged 20–45 years (mean age 28.8 years, SD 4.8 years), registered in the Birth Register during 1982–2013. Table 1 shows the characteristics of the women over six periods in total and by educational level. Mean BMI increased gradually over the study period from 22.7 kg/m^2^ (SD 3.2) to 24.3 kg/m^2^ (SD 4.4) from 1982 to 2013 (Table 1, Fig. 1A). The proportion of women born outside the Nordic countries increased over the study period, from 7.5% in 1982–1988 to 22.2% in 2009–2013. The mean BMI over time among women born in the Nordic counties only can be found in Supplementary Fig. S2.

**Table 1:** Characteristics, age-adjusted mean BMI, and prevalence of BMI in eight categories by period and education.

Period	1982–1988	1989–1993	1994–1998	1999–2003	2004–2008	2009–2013
**All**						
N (% of total)	188,544 (18.4)	101,980 (10.0)	149,639 (14.6)	166,736 (16.3)	195,221 (19.1)	220,210 (21.5)
BMI (kg/m^2^), mean (SD)	22.7 (3.2)	23.1 (3.5)	23.6 (3.8)	24.0 (4.1)	24.1 (4.3)	24.3 (4.4)
Weight (kg), mean (SD)	62.9 (9.7)	64.1 (10.5)	65.6 (11.4)	66.8 (12.3)	67.0 (12.9)	67.3 (13.3)
Height (cm), mean (SD)	166.3 (6.0)	166.5 (6.1)	166.6 (6.2)	166.7 (6.3)	166.6 (6.4)	166.5 (6.5)
Born in Nordic countries (%)	92.5	88.6	86.1	85.2	81.5	77.8
BMI (kg/m^2^), category, n (%)						
16 to < 18.5	7539 (4.0)	4026 (3.9)	4811 (3.2)	4586 (2.8)	5403 (2.8)	6412 (2.9)
18.5 to < 20	22,204 (11.8)	11,744 (11.5)	14,915 (10.0)	14,308 (8.6)	17,349 (8.9)	19,415 (8.8)
20 to < 22.5	75,125 (39.8)	36,801 (36.1)	48,800 (32.6)	50,822 (30.5)	58,560 (30.0)	64,198 (29.2)
22.5 to < 25	49,549 (26.3)	27,033 (26.5)	40,373 (27.0)	45,453 (27.3)	51,735 (26.5)	56,697 (25.7)
25 to < 27.5	18,796 (10.0)	12,235 (12.0)	21,042 (14.1)	25,124 (15.1)	29,094 (14.9)	32,750 (14.9)
27.5 to < 30	8230 (4.4)	5238 (5.1)	9580 (6.4)	12,229 (7.3)	14,778 (7.6)	17,594 (8.0)
30 to < 35	6363 (3.4)	3895 (3.8)	7697 (5.1)	10,250 (6.1)	12,895 (6.6)	16,405 (7.4)
35 to < 60	738 (0.4)	1008 (1.0)	2421 (1.6)	3964 (2.4)	5407 (2.8)	6739 (3.1)
**Educational level**^**a**^						
Missing						
n (%)	1923 (1.0)	313 (0.3)	428 (0.3)	364 (0.2)	891 (0.5)	2307 (1.0)
Lower						
n	28,471 (15.1)	11,893 (11.7)	16,001 (10.7)	14,746 (8.8)	16,420 (8.4)	18,491 (8.4)
BMI (kg/m^2^), mean (SD)	23.1 (3.6)	23.6 (4.0)	24.2 (4.4)	24.6 (4.8)	24.8 (4.9)	25.1 (5.1)
Weight (kg), mean (SD)	63.0 (10.8)	64.3 (12.1)	65.8 (13)	66.9 (14.3)	66.9 (14.6)	67.5 (15.0)
Height (cm), mean (SD)	165.2 (6.3)	165.0 (6.6)	164.9 (6.8)	164.6 (6.9)	164.0 (7.0)	163.9 (6.9)
Born in Nordic countries (%)	87.1	75.5	71.4	66.2	59.2	54.2
BMI (kg/m^2^), category, n (%)						
16 to < 18.5	1524 (5.4)	668 (5.6)	824 (5.1)	732 (5.0)	765 (4.7)	973 (5.3)
18.5 to < 20	3495 (12.3)	1421 (11.9)	1679 (10.5)	1434 (9.7)	1571 (9.6)	1600 (8.7)
20 to < 22.5	10,266 (36.1)	3745 (31.5)	4558 (28.5)	4004 (27.2)	4242 (25.8)	4510 (24.4)
22.5 to < 25	7202 (25.3)	2951 (24.8)	3935 (24.6)	3471 (23.5)	3809 (23.2)	4179 (22.6)
25 to < 27.5	3066 (10.8)	1516 (12.7)	2322 (14.5)	2200 (14.9)	2452 (14.9)	2848 (15.4)
27.5 to < 30	1490 (5.2)	781 (6.6)	1198 (7.5)	1215 (8.2)	1519 (9.3)	1733 (9.4)
30 to < 35	1253 (4.4)	613 (5.2)	1065 (6.7)	1168 (7.9)	1411 (8.6)	1826 (9.9)
35 to < 60	175 (0.6)	198 (1.7)	420 (2.6)	522 (3.5)	651 (4.0)	822 (4.4)
Intermediate						
n	104,920 (55.6)	60,209 (59.0)	83,285 (55.7)	79,138 (47.5)	77,215 (39.6)	79,127 (35.9)
BMI (kg/m^2^), mean (SD)	22.9 (3.3)	23.3 (3.6)	23.9 (4.0)	24.5 (4.4)	24.7 (4.7)	25.0 (4.9)
Weight (kg), mean (SD)	63.2 (10.0)	64.5 (10.8)	66.3 (11.8)	68.0 (13.1)	68.6 (14)	69.1 (14.4)
Height (cm), mean (SD)	166.2 (6.0)	166.5 (6.0)	166.5 (6.1)	166.6 (6.2)	166.4 (6.3)	166.3 (6.4)
Born in Nordic countries (%)	93.9	91.5	88.7	87.4	83.9	82.1
BMI (kg/m^2^), category, n (%)						
16 to < 18.5	4161 (4.0)	2367 (3.9)	2606 (3.1)	2146 (2.7)	2195 (2.8)	2258 (2.9)
18.5 to < 20	11,992 (11.4)	6716 (11.2)	7920 (9.5)	6244 (7.9)	6245 (8.1)	6208 (7.8)
20 to < 22.5	40,434 (38.5)	21,074 (35)	25,860 (31.1)	21,988 (27.8)	20,655 (26.7)	20,134 (25.4)
22.5 to < 25	27,872 (26.6)	15,976 (26.5)	22,162 (26.6)	20,812 (26.3)	19,527 (25.3)	19,458 (24.6)
25 to < 27.5	11,080 (10.6)	7512 (12.5)	12,330 (14.8)	12,805 (16.2)	12,197 (15.8)	12,544 (15.9)
27.5 to < 30	4936 (4.7)	3345 (5.6)	5832 (7.0)	6716 (8.5)	6775 (8.8)	7392 (9.3)
30 to < 35	3976 (3.8)	2563 (4.3)	4965 (6)	5928 (7.5)	6584 (8.5)	7673 (9.7)
35 to < 60	469 (0.4)	656 (1.1)	1610 (1.9)	2499 (3.2)	3037 (3.9)	3460 (4.4)
Higher						
n	53,230 (28.2)	29,565 (29.0)	49,925 (33.4)	72,488 (43.5)	100,695 (51.6)	120,285 (54.6)
BMI (kg/m^2^), mean (SD)	22.4 (2.8)	22.7 (3.1)	23.1 (3.3)	23.5 (3.6)	23.7 (3.8)	23.8 (4)
Weight (kg), mean (SD)	62.6 (8.8)	63.4 (9.5)	64.6 (10.2)	65.7 (11.0)	66.0 (11.7)	66.3 (12.1)
Height (cm), mean (SD)	167.0 (5.8)	167.0 (6.0)	167.1 (6.2)	167.1 (6.2)	167.0 (6.3)	166.9 (6.4)
Born in Nordic countries (%)	93.5	88.8	87.0	86.9	83.8	79.6
BMI (kg/m^2^), category, n (%)						
16 to < 18.5	1751 (3.3)	967 (3.3)	1358 (2.7)	1686 (2.3)	2401 (2.4)	3065 (2.5)
18.5 to < 20	6440 (12.1)	3567 (12.1)	5263 (10.5)	6598 (9.1)	9429 (9.4)	11,411 (9.5)
20 to < 22.5	23,637 (44.4)	11,872 (40.2)	18,248 (36.6)	24,713 (34.1)	33,406 (33.2)	38,978 (32.4)
22.5 to < 25	14,015 (26.3)	8020 (27.1)	14,161 (28.4)	21,078 (29.1)	28,206 (28.0)	32,520 (27)
25 to < 27.5	4489 (8.4)	3176 (10.7)	6334 (12.7)	10,076 (13.9)	14,304 (14.2)	16,988 (14.1)
27.5 to < 30	1736 (3.3)	1100 (3.7)	2527 (5.1)	4267 (5.9)	6412 (6.4)	8246 (6.9)
30 to < 35	1077 (2)	711 (2.4)	1647 (3.3)	3133 (4.3)	4839 (4.8)	6707 (5.6)
35 to < 60	85 (0.2)	152 (0.5)	387 (0.8)	937 (1.3)	1698 (1.7)	2370 (2.0)

*n* number of individuals, *BMI* body mass index, *SD* standard deviation.

^a^Missing data for educational level 6226 (0.6%) women.

**Figure 2:**
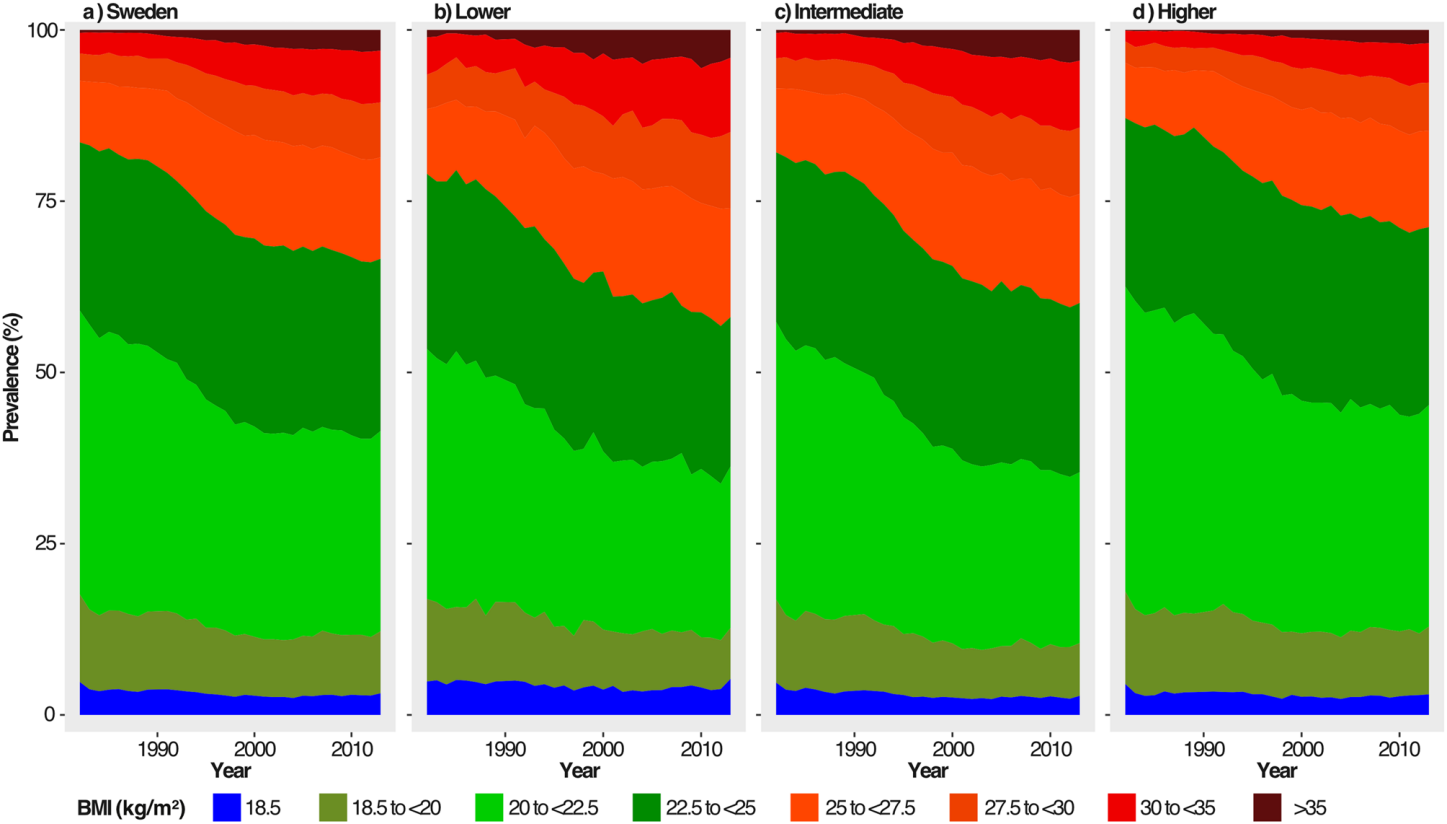
Trends in age-adjusted prevalence of BMI categories. Age-adjusted prevalence of BMI in eight categories from 1982 to 2013. (**a**) All, adjusted for education, (**b**) lower level of education (**c**) intermediate level of education, (**d**) higher level of education. *BMI* body mass index.

**Figure 3:**
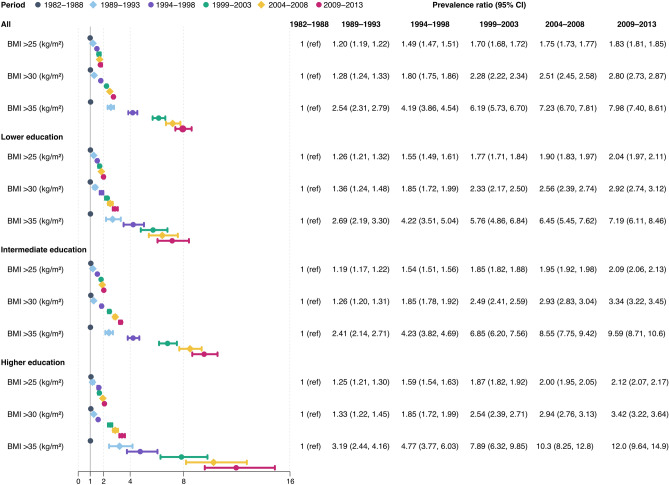
Age-adjusted prevalence ratios of overweight, moderate and severe obesity, by education and period. Age-adjusted prevalence ratios with corresponding confidence intervals for overweight, moderate and severe obesity comparing 1982–1988 versus later periods, in all and stratified by educational level. *CI* confidence intervals, *n* number of individuals, *BMI* body mass index, *ref* reference.

The proportion of women with conventionally defined normal weight (BMI ≥ 18.5 kg/m^2^ and < 25 kg/m^2^) decreased from 77.9 to 63.7%, and the proportion of women with BMI ≥ 20 kg/m^2^ and < 22.5 kg/m^2^ from 39.8 to 29.2%, while the proportion with BMI < 18.5 kg/m^2^ decreased only slightly (Table 1, Fig. 2a). The total prevalence of overweight and obesity (BMI ≥ 25 kg/m^2^) nearly doubled from 18.1 to 33.4%, while that of obesity (BMI ≥ 30 kg/m^2^) and severe obesity (BMI ≥ 35 kg/m^2^) increased from 3.8 and 0.4% in 1982 to 10.5% and 3.1% in 2013, respectively (Table 2). With the first period as a reference (1982, 1988), the PR for obesity increased for each period and was almost three times higher in 2009–2013 (2.80 [2.73, 2.87]), while PR for severe obesity was almost eight times higher during the same period (7.98 [7.40, 8.61]) (Fig. 3).

**Table 2:** Age-adjusted prevalence and prevalence ratios of overweight, moderate and severe obesity, by period and education.

Period	Prevalence (%)				Prevalence ratio (95% CI)		
	All	Lower	Intermediate	Higher	Lower	Intermediate	Higher
**1982–1988**							
n	188,544	28,471	104,920	53,230			
BMI ≥ 25 kg/m^2^	34,127 (18.1)	5984 (21.0)	20,461 (19.5)	7387 (13.9)	1 (ref)	0.93 (0.90, 0.95)	0.66 (0.63, 0.68)
BMI ≥ 30 kg/m^2^	7101 (3.8)	1428 (5.0)	4445 (4.2)	1162 (2.2)	1 (ref)	0.84 (0.80, 0.89)	0.43 (0.40, 0.47)
BMI ≥ 35 kg/m^2^	738 (0.4)	175 (0.6)	469 (0.4)	85 (0.2)	1 (ref)	0.72 (0.60, 0.86)	0.23 (0.17, 0.30)
**1989–1993**							
n	101,980	11,893	60,209	29,565			
BMI ≥ 25 kg/m^2^	22,379 (21.9)	3109 (26.1)	14,076 (23.4)	5139 (17.4)	1 (ref)	0.88 (0.85, 0.92)	0.63 (0.60, 0.66)
BMI ≥ 30 kg/m^2^	4903 (4.8)	811 (6.8)	3219 (5.3)	863 (2.9)	1 (ref)	0.78 (0.72, 0.84)	0.42 (0.38, 0.47)
BMI ≥ 35 kg/m^2^	1008 (1.0)	198 (1.7)	656 (1.1)	152 (0.5)	1 (ref)	0.65 (0.55, 0.76)	0.29 (0.23, 0.36)
**1994–1998**							
n	149,639	16,001	83,285	49,925			
BMI ≥ 25 kg/m^2^	40,740 (27.2)	5005 (31.3)	24,737 (29.7)	10,895 (21.8)	1 (ref)	0.93 (0.90, 0.95)	0.63 (0.60, 0.65)
BMI ≥ 30 kg/m^2^	10,118 (6.8)	1485 (9.3)	6575 (7.9)	2034 (4.1)	1 (ref)	0.84 (0.80, 0.89)	0.42 (0.39, 0.45)
BMI ≥ 35 kg/m^2^	2421 (1.6)	420 (2.6)	1610 (1.9)	387 (0.8)	1 (ref)	0.73 (0.65, 0.81)	0.28 (0.25, 0.33)
**1999–2003**							
n	166,736	14,746	79,138	72,488			
BMI ≥ 25 kg/m^2^	51,567 (30.9)	5105 (34.6)	27,948 (35.3)	18,413 (25.4)	1 (ref)	0.99 (0.96, 1.02)	0.65 (0.63, 0.67)
BMI ≥ 30 kg/m^2^	14,214 (8.5)	1690 (11.5)	8427 (10.6)	4,4070 (5.6)	1 (ref)	0.90 (0.86, 0.95)	0.45 (0.43, 0.48)
BMI ≥ 35 kg/m^2^	3964 (2.4)	522 (3.5)	2499 (3.2)	937 (1.3)	1 (ref)	0.86 (0.78, 0.95)	0.34 (0.30, 0.38)
**2004–2008**							
n	195,221	16,420	77,215	100,695			
BMI ≥ 25 kg/m^2^	62,174 (31.8)	6033 (36.7)	28,593 (37.0)	27,253 (27.1)	1 (ref)	0.97 (0.94, 1.00)	0.63 (0.61, 0.65)
BMI ≥ 30 kg/m^2^	18,302 (9.4)	2062 (12.6)	9621 (12.5)	6537 (6.5)	1 (ref)	0.96 (0.91, 1.01)	0.47 (0.44, 0.49)
BMI ≥ 35 kg/m^2^	5407 (2.8)	651 (4.0)	3037 (3.9)	1698 (1.7)	1 (ref)	0.95 (0.87, 1.03)	0.38 (0.35, 0.42)
**2009–2013**							
n	220,210	18,491	79,127	120,285			
BMI ≥ 25 kg/m^2^	73,488 (33.4)	7229 (39.1)	31,069 (39.3)	34,311 (28.5)	1 (ref)	0.98 (0.96, 1.01)	0.64 (0.62, 0.66)
BMI ≥ 30 kg/m^2^	23,144 (10.5)	2648 (14.3)	11,133 (14.1)	9077 (7.5)	1 (ref)	0.96 (0.92, 1.00)	0.47 (0.45, 0.49)
BMI ≥ 35 kg/m^2^	6739 (3.1)	822 (4.4)	3460 (4.4)	270 (2.0)	1 (ref)	0.96 (0.89, 1.03	0.40 (0.37, 0.43)

Age-adjusted prevalence ratios with corresponding confidence intervals was calculated for overweight, moderate and severe obesity comparing lower versus intermediate and higher level of education within each period.

*CI* confidence intervals, *n* number of individuals, *BMI* body mass index, *ref* reference.

### Trends by education

Mean BMI increased across all educational levels over the study period with the largest absolute increase in those with intermediate education (10–12 years), where mean BMI increased from 22.9 kg/m^2^ (SD 3.3) in 1982 to 25.0 kg/m^2^ (SD 4.9) in 2013 (Table 1). Women with higher education (> 12 years) had the least increase in mean BMI (from 22.4 kg/m^2^ [SD 2.8] to 23.8 kg/m^2^ [SD 4.0]), with a widening gap from 1995 and onwards between women with low and intermediate education compared to women with high education (Table 1, Fig. 1a,b). Visual inspection of the prevalence of BMI categories indicated a continuous decline in the proportion of women with BMI between 20 and < 22.5 kg/m^2^ across all educational levels, while the proportion of women with overweight and moderate and severe obesity more than doubled from 1982 to 2013 (Fig. 2a–d).

There was a marked increase in severe obesity (BMI ≥ 35 kg/m^2^) across all education levels over the study period (Table 2); in women with lower level of education from 1982 to 2013 (from 0.6 to 4.4%) with a PR of 7.19 (95% CI 6.11, 8.46) (Fig. 3). Women with intermediate education displayed an almost a ten-fold increase in severe obesity, from 0.4% in 1982 to 4.4% in 2013 and a PR of 9.59 (95% CI 8.71, 10.6). However, women with higher education, in whom the prevalence of severe obesity was initially extremely low in 1982 (0.2%), had the most marked relative increase (PR = 12.0 [95% CI 9.64, 14.9]), but still the lowest prevalence in 2013 (2.0%), compared to the women with intermediate (4.4%) and low (4.4%) education.

### Trends in BMI by county of residence

Supplementary Figs. S3 and S4 and Supplementary Tables S1 and S2 shows age- and education adjusted mean BMI and prevalence of BMI in eight categories broken down by county in total and by period. The three counties with the only major Swedish cities (> 300,000 inhabitants)—Stockholm, Västra Götaland region (Gothenburg), and Skåne county (Malmö), along with Halland county had the lowest prevalence of obesity (BMI ≥ 30 kg/m^2^) (9.4%, 12.2%, 12.2 and 10.3%, respectively), and the lowest mean BMI in 2013. The highest prevalence of obesity in 2013 was found in Blekinge (16.3%), Västmanland (16.0%), and Västernorrland county (17.0%).

## Discussion

Apart from the expected rise in mean BMI over the past three decades, the main finding of our nationwide study among young/mid-adulthood women were the marked increase in obesity and, in particular, of severe obesity across all educational levels and counties in Sweden. Our study demonstrated an increasing mean BMI in young/mid-adulthood women in Sweden, rising from 22.7 to 24.3 kg/m^2^ over the study period. These findings are consistent with previous studies showing increases in mean BMI at younger ages in Sweden and elsewhere^1,2,16^. We also found that the prevalence of moderate obesity more than doubled during the study period (from 3.4% in 1982 to 7.4% in 2013) and that severe obesity (BMI ≥ 35 kg/m^2^) increased six-fold (from 0.4 to 3.1%).

Social inequalities in moderate and severe obesity among young/mid-adulthood Swedish women are growing, with a marked increased prevalence among those with lower and intermediate education, and those living in sparsely populated counties without major cities. However, the prevalence of obesity increased markedly also among those with higher education al level (> 12 years). Overweight and moderate and severe obesity essentially doubled in all educational levels, with the highest relative increase in severe obesity among those with higher education, who had very low initial levels, and still the lowest final prevalence of about 2%. The proportion of women born outside of the Nordic counties increased during the study period. However, this did not affect the mean BMI in the population over time, as can be seen in Supplementary Fig. S2.

The prevalence of severe obesity in 2013 was highest in some of the counties with mostly rural areas (Blekinge, Gävleborg, and Västernorrland county) with current rates of over 5% among these young/mid-adulthood women. The lowest prevalence of severe obesity was found in counties with major cities. Our findings are consistent with previous research showing a clear association between degree of education and obesity^16–18,24–26^. In particular, one study found this increasing social gradient in overweight/obesity among Swedish men aged 18–25 years during the same period as that of the present study^16^. Hence, similar to past studies, our findings reveal growing social inequalities in both BMI and level of obesity among young/mid-adulthood Swedish women.

Onset of obesity and overweight early in life can have multiple adverse health effects throughout the life course^27^ and are likely to lead to lifelong overweight or obesity^28^. These increasing trends in BMI and obesity, especially severe obesity, in young adulthood are likely to cause a rise in serious health problems, in particular the risk of developing type 2 diabetes, heart failure, and other heart diseases. Obesity, and especially longstanding obesity, is associated with ventricular dysfunction and cardiac remodeling^27,29^. Being obese in youth is associated with atherosclerosis in adulthood, however, mostly due to tracking of body mass from youth to young adulthood^30^. Still, the increase in obesity in the young adults might explain the increased rates of early heart failure^31,32^, coronary heart disease^33^ and ischemic stroke^34^ observed in Sweden and elsewhere, particularly among younger people with low education level^35^. Additionally, we have previously shown that even what is considered normal BMI (BMI 22.5–25 kg/m^2^), compared to being very lean (BMI 20–22.5 kg/m^2^) in young/mid-adulthood is associated with increased risk of developing heart failure, cardiomyopathy, and other heart diseases later in life^10,11,13,14^, indicating rising rates of cardiovascular disease as the currently young and middle-aged progress into an older age, with a potentially widening socioeconomic gap. Additional, obesity in mothers before conception is strongly associated with child obesity^36^. Considering the increasing prevalence of obesity worldwide, this could further exacerbate the obesity trends also in children, especially in socially disadvantaged groups.

A major strength is that this large study with national coverage included nearly all women aged 20–44 years who gave birth to their first child during 1982–2013 (estimated coverage 99%). In addition, we used the LISA database to obtain information on social inequalities, here defined as education level and county of residence, as income could be a less accurate indicator of social status at a younger age. However, there are also some limitations to this study. Weight was estimated for a part of the study period (1982–1989); in addition, height was self-reported, but this is unlikely to have any major impact on the overall pattern that we describe. Moreover, there is no information on anthropometry other than weight or height, e.g., abdominal obesity, However, BMI has been shown to be a reasonably good measure for investigating the trends and prevalence of overweight and obesity over time. Moreover, BMI has been used worldwide in numerous studies and different populations, which enables comparisons. Few women have their first child after the age 45 in Sweden and historically the fraction of women who are childless has decreased from 20% (women born 1910) to 13.5% in women aged 45 year in 1995 (born 1950) with no change at age 45 in women born 1960 and 1970^37^. About 5% of women are voluntary childless^38^. The most common cause of childlessness is physiological or medical factors. Other factors are under or over weight and age. The fraction of childless women is not likely to have increased during the study period, despite the increase in age for women having their first child. Hence, this is potential limitation since other factors (e.g., assisted reproduction) could have counteracted the effect of increasing age and BMI on childlessness during the study period^37^.

In conclusion, both BMI and in particular moderate and severe obesity have increased markedly in young/mid-adulthood Swedish women, with a widening gap in BMI between women with lower and higher education. These growing social inequalities in BMI are likely to cause a rising divide in serious health problems following early and long-lasting obesity. Given the strong association between obesity in youth on risk of early cardiovascular disease, and the considerable tracking of body weight over the life course, this could portend not only a rising trend of early cardiovascular disease, particularly in women with lower level of education, but also a massive increase in cardiovascular disease and diabetes when these birth cohorts progress into the age where cardiovascular disease is common.

## Supplementary Information

Supplementary Information.

## Data Availability

Data are available from the sources stated in the paper on request to the data providers, fulfilling legal and regulatory requirements and with permission from the Swedish Ethical Review Authority.
