# Supplementary material for: Social inequalities and trends in pre-pregnancy body mass index in Swedish women
Source: Sci Rep. 2021 Jun 8;11:12056. doi: 10.1038/s41598-021-91441-7 (PMC8187407; doi:10.1038/s41598-021-91441-7)
Supplement: Supplementary file 1 — Supplementary Information. [file 41598_2021_91441_MOESM1_ESM.docx]

**Social inequalities and trends in pre-pregnancy body mass index in Swedish women**

**Christina E Lundberg, Maria Ryd, Martin Adiels, Annika Rosengren_,_ and Lena Björck**

**Supplementary material:**

**Supplementary Figure S1.** Age distribution at pregnancy by period

**Supplementary Figure S2.** Trends in age-adjusted mean BMI, for women born in the Nordic countries

**Supplementary Figure S3.** Trends in age- and education-adjusted mean BMI by county of residence

**Supplementary Figure S4.** Trends in age- and education adjusted prevalence of BMI categories by county of residence

**Supplementary Table S1.** Age- and education adjusted mean BMI by period and county

**Supplementary Table S2.** Age- and education adjusted prevalence of BMI in eight categories by period and county of residence

**
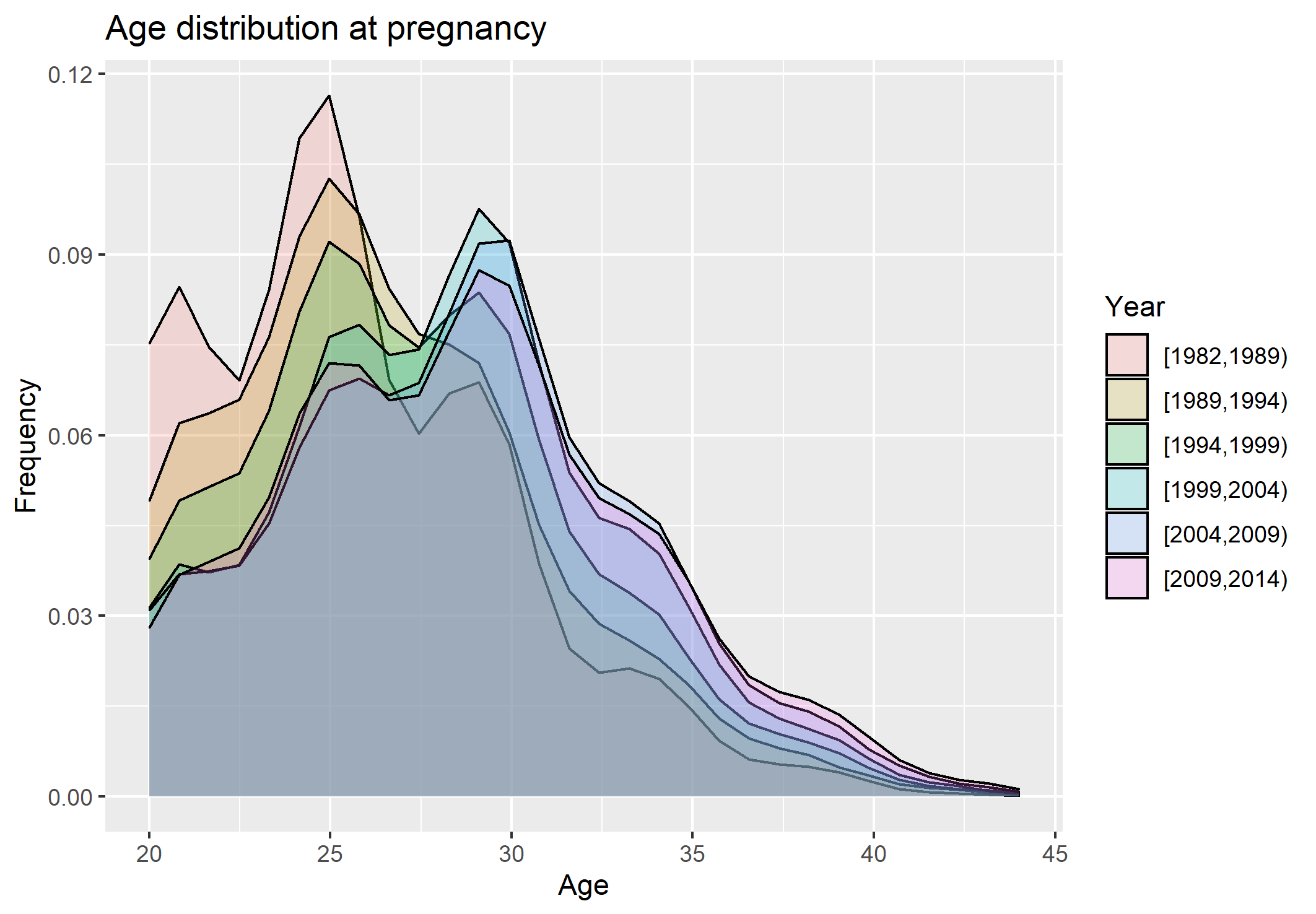
**

**Supplementary Figure S1.** Age distribution at pregnancy by period.

**
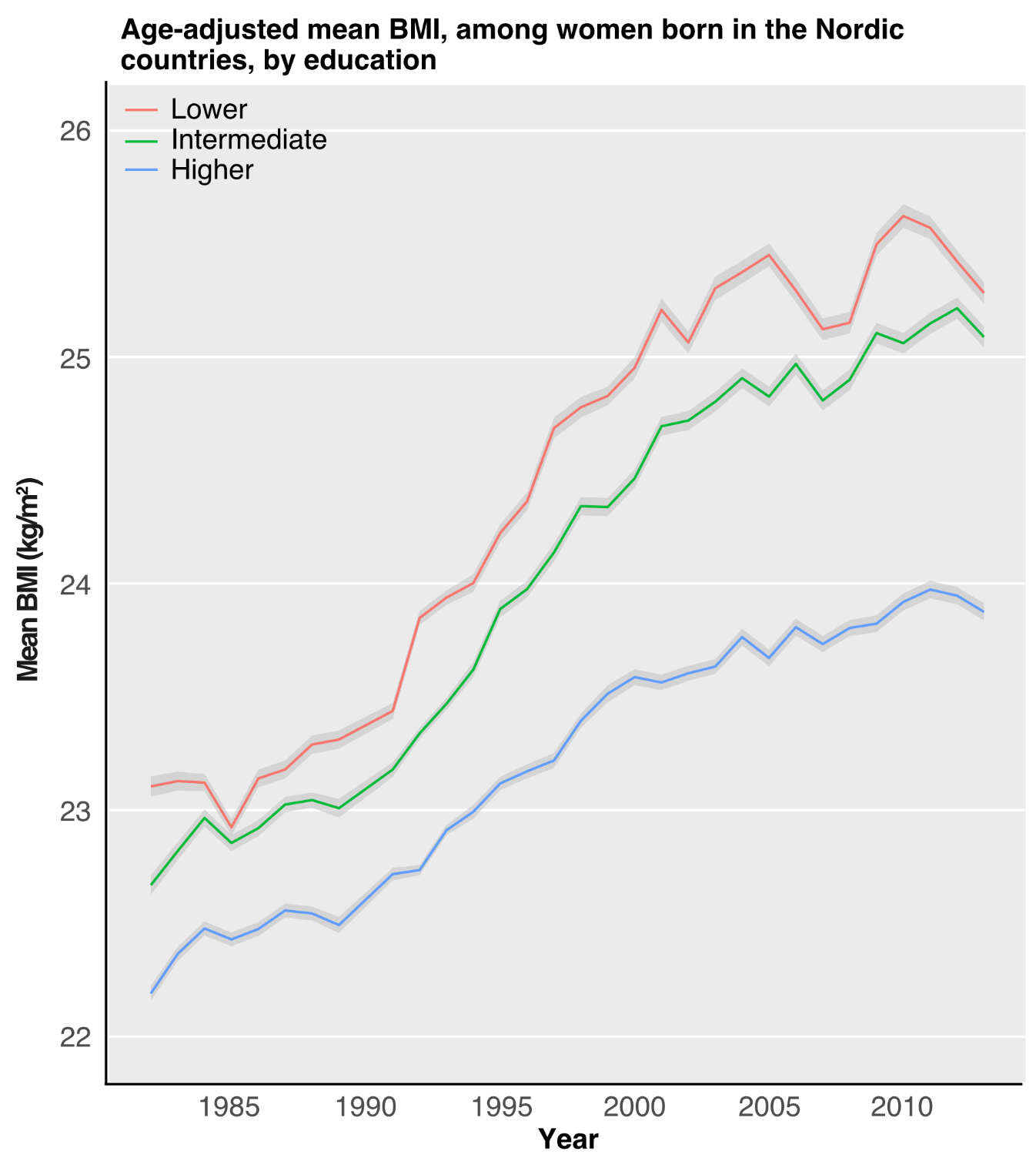
**

**Supplementary Figure S2.** Trends in age-adjusted mean BMI, for women born in the Nordic countries. The lines show the yearly mean estimates and the shades show the 95% confidence intervals from 1982–2013.


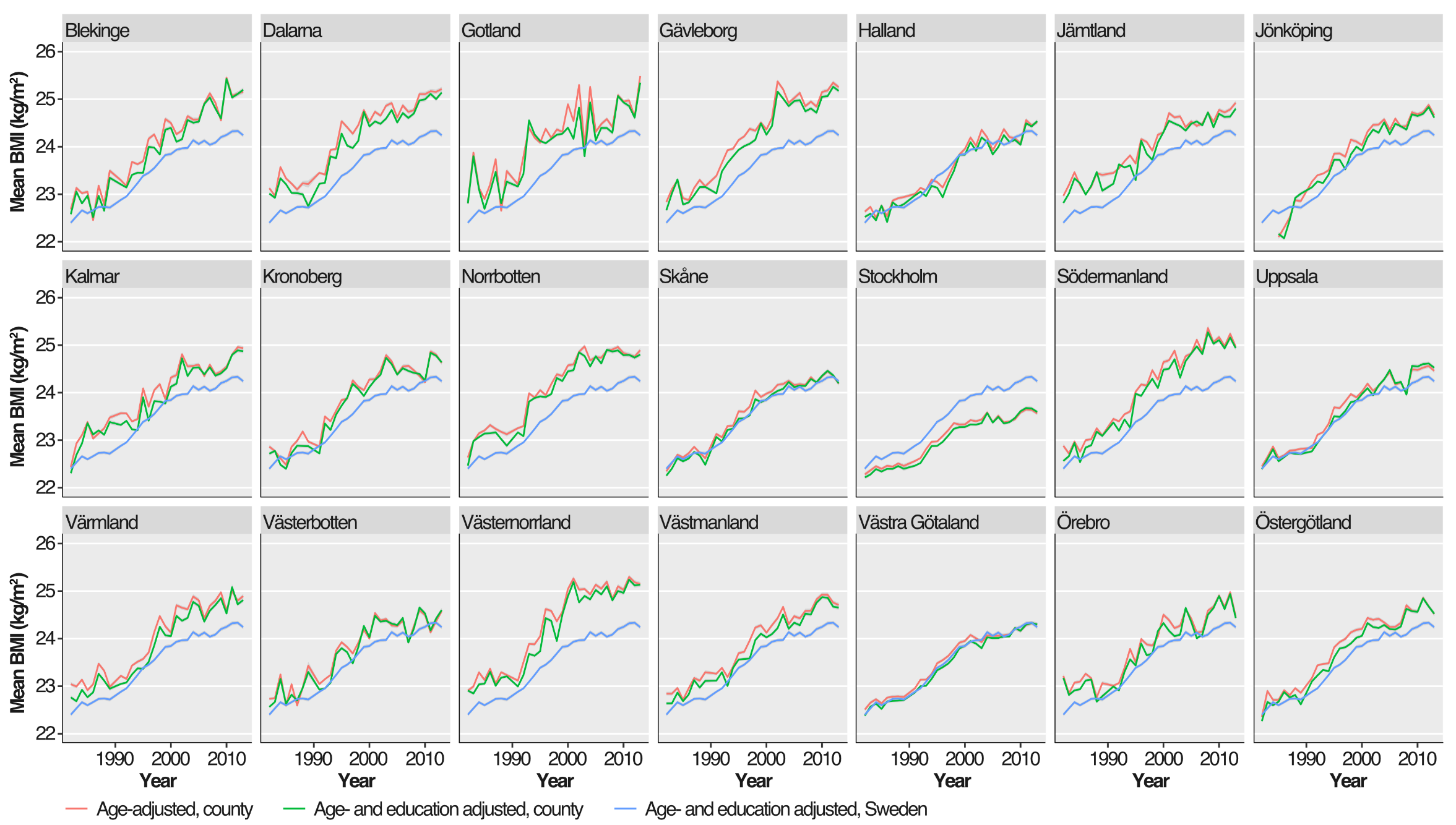


**Supplementary Figure S3.** Trends in age- and education-adjusted mean BMI (kg/m^2^) by county of residence. The lines show the yearly mean estimates from 1982–2013 by county. The blue line represents age- and education-adjusted mean BMI (kg/m^2^) in Sweden, the green and read lines represents age- and age- and education-adjusted mean BMI (kg/m^2^) by county. BMI= body mass index

**
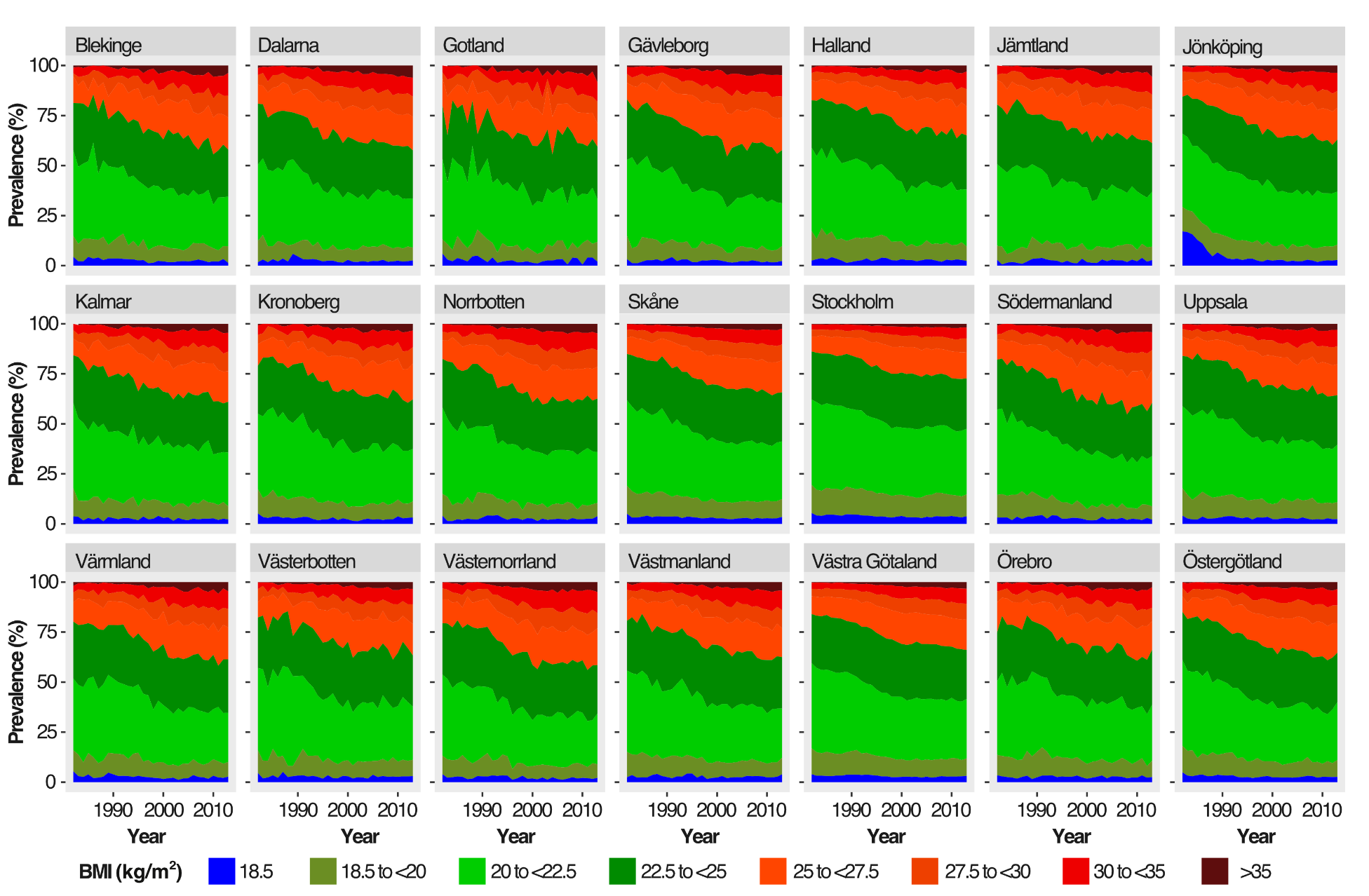
**

**Supplementary Figure S4.** Trends in age- and education adjusted prevalence of BMI (kg/m^2^) categories by county of residence

Age- and education adjusted prevalence of BMI in eight categories from 1982–2013. BMI= body mass index

| **Year** | **1982–1988** | **1989–1993** | **1994–1998** | **1999–2003** | **2004–2008** | **2009–2013** |
| --- | --- | --- | --- | --- | --- | --- |
| **County** | **BMI, kg/m^2^,**  **mean (SD)** | **BMI, kg/m^2^,**  **mean (SD)** | **BMI, kg/m^2^,**  **mean (SD)** | **BMI, kg/m^2^,**  **mean (SD)** | **BMI, kg/m^2^,**  **mean (SD)** | **BMI, kg/m^2^,**  **mean (SD)** |
| Blekinge | 22.8 (3.1) | 23.3 (3.6) | 23.8 (3.7) | 24.3 (4.3) | 24.8 (4.7) | 25.1 (4.8) |
| Dalarna | 23.1 (3.2) | 23.4 (3.5) | 24.0 (3.9) | 24.6 (4.3) | 24.7 (4.6) | 25.0 (5.0) |
| Gotland | 23.1 (3.1) | 23.7 (3.9) | 24.2 (4.0) | 24.3 (4.0) | 24.4 (4.5) | 25.0 (4.9) |
| Gävleborg | 23.0 (3.1) | 23.4 (3.6) | 24.0 (3.9) | 24.7 (4.4) | 24.9 (4.8) | 25.1 (4.7) |
| Halland | 22.6 (3.0) | 23.0 (3.5) | 23.2 (3.6) | 24.0 (4.0) | 24.1 (4.2) | 24.3 (4.4) |
| Jämtland | 23.2 (3.0) | 23.5 (3.7) | 22.7 (3.6) | 24.4 (4.2) | 24.5 (4.5) | 24.6 (4.6) |
| Jönköping | 22.2 (3.6) | 23.2 (3.5) | 23.6 (3.7) | 24.2 (4.1) | 24.4 (4.3) | 24.7 (4.5) |
| Kalmar | 23.0 (3.1) | 23.3 (3.4) | 23.6 (3.7) | 24.3 (4.4) | 24.5 (4.4) | 24.7 (4.6) |
| Kronoberg | 22.7 (3.2) | 23.1 (3.3) | 23.9 (3.9) | 24.3 (4.2) | 24.4 (4.5) | 24.6 (4.6) |
| Norrbotten | 23.0 (3.1) | 23.3 (3.7) | 24.0 (3.9) | 24.6 (4.4) | 24.7 (4.7) | 24.8 (4.8) |
| Skåne | 22.6 (3.1) | 23.0 (3.4) | 23.5 (3.7) | 24.0 (4.1) | 24.2 (4.3) | 24.3 (4.4) |
| Stockholm | 22.4 (3.0) | 22.6 (3.1) | 23.0 (3.4) | 23.3 (3.7) | 23.4 (3.9) | 23.6 (4.1) |
| Södermanland | 22.8 (3.1) | 23.3 (3.5) | 23.9 (3.9) | 24.4 (4.3) | 24.9 (4.6) | 25.0 (4.7) |
| Uppsala | 22.6 (3.0) | 22.9 (3.3) | 23.5 (3.7) | 24.0 (4.1) | 24.3 (4.4) | 24.6 (4.6) |
| Värmland | 22.9 (3.1) | 23.1 (3.3) | 23.7 (3.8) | 24.3 (4.2) | 24.6 (4.6) | 24.8 (4.6) |
| Västerbotten | 22.8 (2.9) | 23.0 (3.3) | 23.7 (3.7) | 24.3 (4.2) | 24.2 (4.3) | 24.5 (4.6) |
| Västernorrland | 23.1 (3.1) | 23.3 (3.4) | 24.0 (4.0) | 24.8 (4.5) | 24.9 (4.6) | 25.1 (4.8) |
| Västmanland | 22.8 (3.1) | 23.1 (3.4) | 23.6 (3.7) | 24.2 (4.1) | 24.4 (4.5) | 24.8 (4.7) |
| Västra Götaland | 22.6 (3.0) | 22.9 (3.3) | 23.4 (3.6) | 23.9 (4.0) | 24.0 (4.2) | 24.3 (4.4) |
| Örebro | 23.0 (3.2) | 23.0 (3.3) | 23.6 (3.8) | 24.2 (4.2) | 24.3 (4.5) | 24.7 (4.6) |
| Östergötland | 22.7 (3.1) | 23.2 (3.5) | 23.7 (3.7) | 24.2 (4.1) | 24.3 (4.3) | 24.6 (4.5) |

**Supplementary Table S1.** Age- and education adjusted mean BMI (kg/m^2^) by period and county.

SD= standard deviation

| **Year** | **1982–1988** | **1989–1993** | **1994–1998** | **1999–2003** | **2004–2008** | **2009–2013** |
| --- | --- | --- | --- | --- | --- | --- |
| **County** | **n %** | **n %** | **n %** | **n %** | **n %** | **n %** |
| **BMI (kg/m^2^) category 16 to <18.5** | | | | | | |
| Blekinge | 130 (3.9) | 70 (4.0) | 63 (2.6) | 67 (2.4) | 75 (2.6) | 73 (2.5) |
| Dalarna | 104 (2.7) | 107 (3.9) | 104 (2.5) | 83 (1.9) | 131 (2.7) | 146 (2.9) |
| Gotland | 44 (3.8) | 18 (3.1) | 20 (2.3) | 23 (2.6) | 35 (4.1) | 40 (3.9) |
| Gävleborg | 122 (2.6) | 81 (2.9) | 93 (2.2) | 101 (2.3) | 121 (2.5) | 106 (2.0) |
| Halland | 192 (3.5) | 89 (2.9) | 177 (4.0) | 198 (3.8) | 158 (2.7) | 200 (3.3) |
| Jämtland | 25 (1.3) | 48 (3.8) | 31 (1.6) | 43 (2.1) | 53 (2.2) | 81 (3.2) |
| Jönköping | 871 (11) | 150 (3.9) | 154 (2.7) | 180 (2.9) | 212 (3.0) | 214 (2.8) |
| Kalmar | 152 (2.8) | 76 (2.8) | 101 (2.8) | 125 (3.5) | 116 (2.8) | 144 (3.1) |
| Kronoberg | 175 (4.1) | 69 (3.2) | 78 (2.5) | 71 (2.1) | 115 (3.1) | 117 (3.1) |
| Norrbotten | 130 (2.4) | 124 (4.3) | 100 (2.5) | 126 (3.3) | 87 (2.2) | 135 (3.1) |
| Skåne | 1,005 (3.9) | 490 (3.8) | 713 (3.7) | 660 (3.0) | 822 (3.0) | 959 (3.2) |
| Stockholm | 1,621 (4.8) | 1,076 (4.7) | 1,236 (3.6) | 1476 (3.6) | 1713 (3.6) | 2,254 (3.8) |
| Södermanland | 204 (3.5) | 116 (3.9) | 133 (3.2) | 109 (2.5) | 101 (2.4) | 133 (2.7) |
| Uppsala | 237 (2.8) | 135 (3.4) | 188 (3.3) | 168 (2.9) | 162 (2.7) | 184 (2.4) |
| Värmland | 165 (3.3) | 108 (3.9) | 136 (3.2) | 83 (2.0) | 157 (3.2) | 141 (3.4) |
| Västerbotten | 60 (2.8) | 92 (3.7) | 89 (2.3) | 145 (3.7) | 175 (3.2) | 168 (3.1) |
| Västernorrland | 161 (2.7) | 72 (3.3) | 99 (3.3) | 59 (1.9) | 100 (2.3) | 89 (2.1) |
| Västmanland | 157 (2.9) | 75 (2.7) | 144 (3.7) | 111 (2.5) | 139 (2.9) | 182 (3.4) |
| Västra Götaland | 1,229 (3.5) | 652 (3.9) | 816 (3.3) | 843 (3.0) | 929 (2.7) | 1,191 (3.3) |
| Örebro | 126 (2.5) | 85 (2.9) | 145 (3.4) | 124 (2.8) | 157 (2.9) | 188 (3.0) |
| Östergötland | 7,335 (3.9) | 3,900 (3.8) | 4,835 (3.2) | 4983 (3.0) | 5852 (3.0) | 6,950 (3.2) |
| **BMI (kg/m^2^) category 18.5 to <20** | | | | | | |
| Blekinge | 323 (9.6) | 155 (8.9) | 187 (7.7) | 226 (8.2) | 210 (7.3) | 168 (5.8) |
| Dalarna | 359 (9.1) | 223 (8.2) | 316 (7.4) | 278 (6.5) | 406 (8.4) | 358 (7.0) |
| Gotland | 88 (7.6) | 64 (11.0) | 81 (9.3) | 51 (5.7) | 61 (7.3) | 81 (7.9) |
| Gävleborg | 497 (10.4) | 230 (8.3) | 320 (7.5) | 284 (6.6) | 322 (6.5) | 374 (7.2) |
| Halland | 672 (12.3) | 352 (11.4) | 464 (10.5) | 435 (8.4) | 498 (8.5) | 449 (7.3) |
| Jämtland | 137 (6.8) | 131 (10.3) | 171 (8.8) | 146 (7.0) | 154 (6.4) | 186 (7.3) |
| Jönköping | 907 (11.8) | 400 (10.5) | 536 (9.3) | 519 (8.4) | 537 (7.6) | 562 (7.3) |
| Kalmar | 540 (10.0) | 226 (8.27) | 342 (9.5) | 243 (6.8) | 321 (7.8) | 327 (7.2) |
| Kronoberg | 474 (11.1) | 230 (10.7) | 242 (7.8) | 261 (7.7) | 329 (8.8) | 263 (7.0) |
| Norrbotten | 539 (9.8) | 298 (10.4) | 313 (7.9) | 261 (6.8) | 315 (7.9) | 283 (6.5) |
| Skåne | 3,145 (12.1) | 1,389 (10.8) | 1,759 (9.1) | 1907 (8.5) | 2330 (8.5) | 2,497 (8.2) |
| Stockholm | 4,343 (12.8) | 2,967 (12.8) | 3,879 (11.4) | 4153 (10.2) | 5020 (10.6) | 5,820 (9.7) |
| Södermanland | 677 (11.5) | 311 (10.6) | 348 (8.5) | 336 (7.7) | 287 (6.9) | 328 (6.6) |
| Uppsala | 974 (11.4) | 406 (10.1) | 545 (9.5) | 454 (7.9) | 516 (8.6) | 528 (6.9) |
| Värmland | 472 (9.5) | 258 (9.4) | 408 (9.7) | 290 (6.9) | 344 (7.0) | 290 (6.9) |
| Västerbotten | 223 (10.4) | 247 (10.0) | 318 (8.4) | 258 (6.4) | 394 (7.3) | 437 (8.0) |
| Västernorrland | 547 (9.2) | 181 (8.2) | 205 (6.9) | 217 (7.1) | 252 (5.7) | 282 (6.5) |
| Västmanland | 586 (10.7) | 262 (9.5) | 358 (9.1) | 360 (8.2) | 371 (7.7) | 373 (7.0) |
| Västra Götaland | 4,050 (11.4) | 1,900 (11.4) | 2,382 (9.6) | 2521 (8.9) | 2815 (8.1) | 3,001 (8.3) |
| Örebro | 526 (10.6) | 290 (9.9) | 395 (9.4) | 340 (7.6) | 447 (8.3) | 468 (7.4) |
| Östergötland | 984 (10.5) | 530 (10.5) | 610 (8.4) | 552 (7.0) | 702 (7.8) | 686 (7.2) |
| **BMI (kg/m^2^) category 20 to <22.5** | | | | | | |
| Blekinge | 1,306 (38.8) | 623 (35.7) | 724 (29.6) | 722 (26.1) | 728 (25.2) | 687 (23.8) |
| Dalarna | 1,443 (36.7) | 868 (31.7) | 1,171 (27.5) | 1138 (26.6) | 1218 (25.3) | 1,161 (22.8) |
| Gotland | 428 (37.0) | 184 (31.9) | 252 (29.1) | 214 (24.1) | 208 (24.7) | 249 (24.1) |
| Gävleborg | 1,860 (39.1) | 884 (31.8) | 1,209 (28.5) | 1060 (24.5) | 1217 (24.6) | 1,193 (22.8) |
| Halland | 2,174 (39.7) | 1,148 (37.3) | 1,430 (32.3) | 1394 (26.8) | 1682 (28.8) | 1,663 (27.2) |
| Jämtland | 804 (39.8) | 384 (30.4) | 597 (30.9) | 554 (26.5) | 664 (27.7) | 686 (27.0) |
| Jönköping | 2,712 (35.2) | 1,293 (33.7) | 1,747 (30.2) | 1635 (26.4) | 1852 (26.0) | 1,909 (24.9) |
| Kalmar | 2,025 (37.5) | 918 (33.6) | 1,063 (29.7) | 1047 (29.4) | 1111 (26.8) | 1,095 (23.9) |
| Kronoberg | 1,724 (40.2) | 761 (35.4) | 978 (31.6) | 952 (28.1) | 1000 (26.7) | 949 (25.3) |
| Norrbotten | 2,048 (37.4) | 903 (31.6) | 1,126 (28.4) | 1013 (26.4) | 1016 (25.4) | 1,095 (25.0) |
| Skåne | 10,365 (40.0) | 4,591 (35.8) | 6,150 (31.8) | 6447 (28.8) | 7684 (28.1) | 8,052 (26.5) |
| Stockholm | 14,050 (41.2) | 8,594 (37.1) | 11,721 (34.6) | 13,083 (32.0) | 14,810 (31.3) | 18,200 (30.3) |
| Södermanland | 2,217 (37.7) | 912 (31.1) | 1,139 (27.8) | 1,168 (26.8) | 996 (23.8) | 1,151 (23.2) |
| Uppsala | 3,486 (40.7) | 1,429 (35.6) | 1,671 (29.0) | 1,608 (27.8) | 1,580 (26.2) | 2,011 (26.1) |
| Värmland | 1,825 (36.6) | 902 (32.7) | 1,277 (30.5) | 1,165 (27.7) | 1,271 (26.0) | 1,009 (24.0) |
| **Year** | **1982–1988** | **1989–1993** | **1994–1998** | **1999–2003** | **2004–2008** | **2009–2013** |
| **County** | **n %** | **n %** | **n %** | **n %** | **n %** | **n %** |
| **BMI (kg/m^2^) category 20 to <22.5** | | | | | | |
| Västerbotten | 878 (41.1) | 848 (34.4) | 1,177 (30.9) | 1,115 (28.4) | 1,468 (27.1) | 1,388 (25.4) |
| Västernorrland | 2,167 (36.5) | 721 (32.7) | 839 (28.1) | 774 (25.4) | 1,139 (25.7) | 978 (22.7) |
| Västmanland | 2,141 (39.1) | 985 (35.9) | 1,207 (30.6) | 1,212 (27.6) | 1,259 (26.1) | 1,261 (23.5) |
| Västra Götaland | 14,378 (40.3) | 5,918 (35.5) | 7,840 (31.5) | 8,433 (29.8) | 9,809 (28.3) | 9,882 (27.4) |
| Örebro | 1,810 (36.4) | 1,040 (35.4) | 1,238 (29.3) | 1,283 (28.7) | 1,467 (27.1) | 1,542 (24.5) |
| Östergötland | 3,708 (39.5) | 1,761 (34.8) | 2,213 (30.4) | 2,144 (27.0) | 2,339 (25.8) | 2,298 (23.9) |
| **BMI (kg/m^2^) category 22.5 to <25** | | | | | | |
| Blekinge | 941 (28.0) | 400 (22.9) | 651 (26.6) | 777 (28.0) | 709 (24.6) | 697 (24.2) |
| Dalarna | 1,082 (27.6) | 747 (27.3) | 1,155 (27.2) | 1,094 (25.6) | 1,223 (25.4) | 1,289 (25.3) |
| Gotland | 333 (28.8) | 169 (29.3) | 237 (27.3) | 270 (30.4) | 208 (24.7) | 245 (23.8) |
| Gävleborg | 1,308 (27.5) | 763 (27.4) | 1,169 (27.5) | 1,176 (27.1) | 1,224 (24.8) | 1,270 (24.3) |
| Halland | 1,443 (26.4) | 850 (27.6) | 1,184 (26.7) | 1,390 (26.7) | 1,540 (26.4) | 1,612 (26.4) |
| Jämtland | 613 (30.3) | 376 (29.8) | 528 (27.3) | 553 (26.4) | 559 (23.3) | 650 (25.6) |
| Jönköping | 1,853 (24.1) | 1,039 (27.1) | 1,559 (27.0) | 1,663 (26.8) | 1,929 (27.1) | 1,895 (24.7) |
| Kalmar | 1,571 (29.1) | 820 (30.0) | 930 (25.9) | 922 (25.9) | 1,046 (25.2) | 1,152 (25.2) |
| Kronoberg | 1,078 (25.1) | 617 (28.7) | 825 (26.7) | 896 (26.4) | 897 (23.9) | 861 (23.0) |
| Norrbotten | 1,556 (28.4) | 763 (26.7) | 1,123 (28.3) | 905 (23.6) | 957 (24.0) | 1,136 (25.9) |
| Skåne | 6,717 (25.9) | 3,393 (26.5) | 5,149 (26.6) | 5,953 (26.6) | 6861 (25.1) | 7,561 (24.9) |
| Stockholm | 8,554 (25.1) | 5,960 (25.8) | 8,731 (25.7) | 10,831 (26.5) | 12,104 (25.6) | 14,969 (24.9) |
| Södermanland | 1,573 (26.7) | 797 (27.2) | 1,134 (27.6) | 1,175 (26.9) | 1,053 (25.2) | 1,199 (24.2) |
| Uppsala | 2,288 (26.7) | 1,117 (27.9) | 1,631 (28.3) | 1,508 (26.1) | 1,508 (25.1) | 1,927 (25.0) |
| Värmland | 1,375 (27.6) | 772 (28.0) | 1,079 (25.7) | 1,078 (25.6) | 1,240 (25.3) | 1,093 (26.0) |
| Västerbotten | 580 (27.2) | 694 (28.2) | 1,019 (26.7) | 977 (24.9) | 1,400 (25.9) | 1,382 (25.3) |
| Västernorrland | 1,777 (29.9) | 637 (28.9) | 827 (27.7) | 776 (25.4) | 1,060 (23.9) | 1,088 (25.2) |
| Västmanland | 1,437 (26.3) | 740 (27.0) | 1,009 (25.6) | 1,208 (27.5) | 1,179 (24.4) | 1327 (24.7) |
| Västra Götaland | 9,585 (26.9) | 4,446 (26.7) | 6,953 (28.0) | 7,475 (26.4) | 9,169 (26.5) | 8,954 (24.8) |
| Örebro | 1,406 (28.3) | 836 (28.4) | 1,119 (26.5) | 1,198 (26.8) | 1,397 (25.8) | 1,531 (24.3) |
| Östergötland | 2,547 (27.1) | 1,347 (26.6) | 2,065 (28.4) | 2,081 (26.2) | 2,258 (24.9) | 2,371 (24.7) |
| **BMI (kg/m^2^) category 25 to 27.5** | | | | | | |
| Blekinge | 366 (10.9) | 257 (14.7) | 418 (17.1) | 426 (15.4) | 480 (16.6) | 458 (16.0) |
| Dalarna | 494 (12.6) | 395 (14.5) | 711 (16.7) | 732 (17.1) | 760 (15.8) | 856 (16.8) |
| Gotland | 136 (11.7) | 59 (10.2) | 139 (16.0) | 170 (19.1) | 99 (11.8) | 141 (13.7) |
| Gävleborg | 502 (10.6) | 398 (14.3) | 710 (16.7) | 762 (17.6) | 912 (18.5) | 944 (18.1) |
| Halland | 540 (9.87) | 362 (11.8) | 581 (13.1) | 937 (18.0) | 885 (15.2) | 995 (16.3) |
| Jämtland | 232 (11.5) | 165 (13.1) | 310 (16.0) | 359 (17.1) | 402 (16.7) | 376 (14.8) |
| Jönköping | 747 (9.70) | 550 (14.4) | 906 (15.7) | 1,043 (16.8) | 1,203 (16.9) | 1,161 (15.1) |
| Kalmar | 580 (10.7) | 389 (14.2) | 554 (15.5) | 542 (15.2) | 684 (16.5) | 765 (16.7) |
| Kronoberg | 437 (10.2) | 241 (11.2) | 475 (15.4) | 499 (14.7) | 631 (16.8) | 676 (18.0) |
| Norrbotten | 674 (12.3) | 400 (14.0) | 683 (17.3) | 591 (15.4) | 635 (15.9) | 665 (15.2) |
| Skåne | 2,413 (9.32) | 1,571 (12.3) | 2,757 (14.3) | 3,517 (15.7) | 4,349 (15.9) | 4,875 (16.0) |
| Stockholm | 3,043 (8.93) | 2,612 (11.3) | 4,570 (13.5) | 5,740 (14.1) | 6,571 (13.9) | 8,698 (14.5) |
| Södermanland | 653 (11.1) | 442 (15.1) | 685 (16.7) | 722 (16.5) | 739 (17.7) | 829 (16.7) |
| Uppsala | 866 (10.1) | 486 (12.1) | 872 (15.2) | 947 (16.4) | 834 (13.8) | 1,239 (16.1) |
| Värmland | 624 (12.5) | 377 (13.7) | 615 (14.7) | 720 (17.1) | 840 (17.2) | 663 (15.8) |
| Västerbotten | 211 (9.9) | 281 (11.4) | 641 (16.8) | 660 (16.8) | 819 (15.1) | 789 (14.4) |
| Västernorrland | 695 (11.7) | 347 (15.7) | 482 (16.1) | 518 (17.0) | 726 (16.4) | 714 (16.6) |
| Västmanland | 582 (10.6) | 341 (12.4) | 604 (15.3) | 618 (14.1) | 824 (17.1) | 859 (16.0) |
| Västra Götaland | 3524 (9.9) | 1,997 (12.0) | 3,519 (14.2) | 4,362 (15.4) | 5,448 (15.7) | 5,504 (15.2) |
| Örebro | 597 (12.0) | 358 (12.2) | 619 (14.7) | 633 (14.2) | 836 (15.4) | 1,020 (16.2) |
| Östergötland | 943 (10.0) | 692 (13.7) | 1,091 (15.0) | 1,324 (16.7) | 1,455 (16.1) | 1,676 (17.5) |
| **BMI (kg/m^2^) category 27.5 to <30** | | | | | | |
| Blekinge | 156 (4.6) | 105 (6.0) | 191 (7.8) | 200 (7.2) | 233 (8.1) | 332 (11.5) |
| Dalarna | 215 (5.5) | 177 (6.5) | 369 (8.7) | 409 (9.6) | 479 (9.9) | 501 (9.8) |
| Gotland | 67 (5.8) | 43 (7.4) | 75 (8.6) | 68 (7.7) | 131 (15.5) | 115 (11.2) |
| Gävleborg | 245 (5.2) | 213 (7.7) | 364 (8.6) | 335 (7.7) | 398 (8.1) | 528 (10.1) |
| Halland | 249 (4.6) | 117 (3.8) | 286 (6.5) | 384 (7.4) | 461 (7.9) | 566 (9.3) |
| Jämtland | 124 (6.1) | 88 (7.01) | 155 (8.0) | 199 (9.5) | 225 (9.4) | 202 (7.9) |
| Jönköping | 347 (4.5) | 200 (5.2) | 394 (6.8) | 523 (8.4) | 614 (8.6) | 759 (9.9) |
| Kalmar | 270 (5.0) | 151 (5.5) | 295 (8.2) | 279 (7.9) | 339 (8.2) | 424 (9.3) |
| **Year** | **1982–1988** | **1989–1993** | **1994–1998** | **1999–2003** | **2004–2008** | **2009–2013** |
| **County** | **n %** | **n %** | **n %** | **n %** | **n %** | **n %** |
| **BMI (kg/m^2^) category 27.5 to <30** | | | | | | |
| Kronoberg | 221 (5.1) | 118 (5.5) | 235 (7.6) | 270 (8.0) | 292 (7.8) | 350 (9.3) |
| Norrbotten | 260 (4.7) | 189 (6.6) | 304 (7.7) | 373 (9.7) | 382 (9.6) | 426 (9.7) |
| Skåne | 1,239 (4.8) | 701 (5.5) | 1,428 (7.4) | 1,823 (8.2) | 2,306 (8.4) | 2,745 (9.0) |
| Stockholm | 1,319 (3.9) | 1,010 (4.4) | 1,890 (5.6) | 2,647 (6.5) | 3,349 (7.1) | 4,498 (7.5) |
| Södermanland | 298 (5.1) | 199 (6.8) | 314 (7.6) | 385 (8.8) | 392 (9.4) | 553 (11.2) |
| Uppsala | 415 (4.8) | 254 (6.3) | 408 (7.1) | 489 (8.5) | 588 (9.8) | 738 (9.6) |
| Värmland | 265 (5.3) | 156 (5.7) | 271 (6.5) | 396 (9.4) | 415 (8.5) | 409 (9.7) |
| Västerbotten | 103 (4.8) | 139 (5.6) | 284 (7.5) | 319 (8.1) | 459 (8.5) | 557 (10.2) |
| Västernorrland | 324 (5.5) | 142 (6.5) | 255 (8.5) | 272 (8.9) | 483 (10.9) | 430 (10.0) |
| Västmanland | 299 (5.5) | 196 (7.2) | 320 (8.1) | 372 (8.5) | 412 (8.5) | 504 (9.4) |
| Västra Götaland | 1,544 (4.3) | 901 (5.4) | 1,612 (6.5) | 2,155 (7.6) | 2,875 (8.3) | 3,169 (8.8) |
| Örebro | 248 (5.0) | 185 (6.3) | 342 (8.1) | 417 (9.4) | 454 (8.4) | 613 (9.7) |
| Östergötland | 440 (4.7) | 260 (5.1) | 484 (6.7) | 699 (8.8) | 942 (10.4) | 925 (9.6) |
| **BMI (kg/m^2^) category 30 to <35** | | | | | | |
| Blekinge | 133 (4.0) | 96 (5.5) | 140 (5.7) | 231 (8.3) | 305 (10.6) | 313 (10.9) |
| Dalarna | 204 (5.2) | 173 (6.3) | 306 (7.2) | 371 (8.7) | 434 (9.0) | 524 (10.3) |
| Gotland | 59 (5.1) | 30 (5.2) | 44 (5.1) | 57 (6.4) | 61 (7.3) | 95 (9.2) |
| Gävleborg | 195 (4.1) | 156 (5.6) | 290 (6.8) | 446 (10.3) | 413 (8.4) | 545 (10.4) |
| Halland | 176 (3.2) | 124 (4.0) | 221 (5.0) | 336 (6.4) | 415 (7.1) | 460 (7.5) |
| Jämtland | 81 (4.0) | 48 (3.8) | 99 (5.1) | 156 (7.5) | 238 (9.9) | 270 (10.6) |
| Jönköping | 231 (3.0) | 151 (3.9) | 383 (6.6) | 439 (7.1) | 544 (7.7) | 880 (11.5) |
| Kalmar | 213 (3.9) | 104 (3.8) | 226 (6.3) | 276 (7.8) | 372 (9.0) | 453 (9.9) |
| Kronoberg | 165 (3.8) | 98 (4.6) | 190 (6.2) | 335 (9.9) | 328 (8.7) | 352 (9.4) |
| Norrbotten | 247 (4.5) | 136 (4.8) | 229 (5.8) | 390 (10.2) | 420 (10.5) | 428 (9.8) |
| Skåne | 893 (3.5) | 482 (3.8) | 1,013 (5.2) | 1,486 (6.6) | 2,023 (7.4) | 2,628 (8.6) |
| Stockholm | 1,016 (3.0) | 730 (3.2) | 1,462 (4.3) | 2,141 (5.2) | 2,700 (5.7) | 4,127 (6.9) |
| Södermanland | 229 (3.9) | 125 (4.3) | 262 (6.4) | 322 (7.4) | 430 (10.3) | 556 (11.2) |
| Uppsala | 266 (3.1) | 141 (3.5) | 330 (5.7) | 416 (7.2) | 609 (10.1) | 751 (9.8) |
| Värmland | 210 (4.2) | 152 (5.5) | 280 (6.7) | 329 (7.8) | 455 (9.2) | 381 (9.1) |
| Västerbotten | 71 (3.3) | 149 (6.0) | 226 (5.9) | 307 (7.8) | 494 (9.1) | 500 (9.2) |
| Västernorrland | 233 (3.9) | 81 (3.7) | 219 (7.3) | 271 (8.9) | 466 (10.5) | 517 (12.0) |
| Västmanland | 237 (4.3) | 117 (4.3) | 234 (5.9) | 393 (9.0) | 448 (9.3) | 549 (10.2) |
| Västra Götaland | 1,188 (3.3) | 667 (4.0) | 1,307 (5.3) | 1,722 (6.1) | 2,502 (7.2) | 3,043 (8.4) |
| Örebro | 241 (4.8) | 120 (4.1) | 281 (6.7) | 327 (7.3) | 466 (8.6) | 690 (11.0) |
| Östergötland | 372 (4.0) | 230 (4.5) | 480 (6.6) | 560 (7.1) | 730 (8.1) | 927 (9.7) |
| **BMI (kg/m^2^) category 35 to <60** | | | | | | |
| Blekinge | 14 (0.4) | 39 (2.2) | 72 (3.0) | 122 (4.4) | 146 (5.0) | 156 (5.4) |
| Dalarna | 26 (0.7) | 48 (1.7) | 119 (2.8) | 179 (4.2) | 173 (3.6) | 260 (5.1) |
| Gotland | 2 (0.2) | 11 (1.8) | 19 (2.2) | 35 (4.0) | 39 (4.7) | 65 (6.3) |
| Gävleborg | 29 (0.6) | 56 (2.0) | 91 (2.1) | 170 (3.9) | 332 (6.7) | 272 (5.2) |
| Halland | 25 (0.5) | 36 (1.2) | 85 (1.9) | 136 (2.6) | 199 (3.4) | 169 (2.8) |
| Jämtland | 5 (0.2) | 22 (1.8) | 42 (2.2) | 84 (4.0) | 107 (4.5) | 93 (3.7) |
| Jönköping | 30 (0.4) | 49 (1.3) | 105 (1.8) | 192 (3.1) | 224 (3.1) | 288 (3.8) |
| Kalmar | 51 (0.9) | 47 (1.7) | 75 (2.1) | 124 (3.5) | 159 (3.8) | 220 (4.8) |
| Kronoberg | 20 (0.5) | 19 (0.9) | 69 (2.2) | 105 (3.1) | 160 (4.3) | 177 (4.7) |
| Norrbotten | 28 (0.5) | 45 (1.6) | 83 (2.1) | 182 (4.7) | 184 (4.6) | 221 (5.0) |
| Skåne | 121 (0.5) | 204 (1.6) | 389 (2.0) | 582 (2.6) | 995 (3.6) | 1,109 (3.6) |
| Stockholm | 140 (0.4) | 189 (0.8) | 434 (1.3) | 768 (1.9) | 1,028 (2.2) | 1,505 (2.5) |
| Södermanland | 38 (0.7) | 29 (1.0) | 90 (2.2) | 148 (3.4) | 185 (4.4) | 203 (4.1) |
| Uppsala | 36 (0.4) | 45 (1.1) | 115 (2.0) | 188 (3.3) | 224 (3.7) | 320 (4.2) |
| Värmland | 52 (1.0) | 34 (1.2) | 125 (3.0) | 148 (3.5) | 174 (3.6) | 213 (5.1) |
| Västerbotten | 10 (0.5) | 15 (0.6) | 60 (1.6) | 145 (3.7) | 200 (3.7) | 243 (4.5) |
| Västernorrland | 35 (0.6) | 25 (1.1) | 62 (2.1) | 163 (5.4) | 208 (4.7) | 215 (5.0) |
| Västmanland | 32 (0.6) | 27 (1.0) | 67 (1.7) | 118 (2.7) | 199 (4.1) | 308 (5.8) |
| Västra Götaland | 154 (0.4) | 201 (1.2) | 428 (1.7) | 800 (2.8) | 1,092 (3.2) | 1,372 (3.8) |
| Örebro | 14 (0.3) | 29 (1.0) | 79 (1.9) | 141 (3.2) | 193 (3.6) | 246 (3.9) |
| Östergötland | 42 (0.5) | 70 (1.4) | 133 (1.8) | 339 (4.3) | 332 (3.7) | 459 (4.8) |

**Supplementary Table S2.** Age- and education-adjusted prevalence of BMI in eight categories by period and county of residence. Age-adjusted to the year 2013 and adjusted for education. BMI = body mass index, n = number of individuals.
